# 5-(2-Methoxy­benz­yl)-4-(2-methoxy­phen­yl)-4*H*-1,2,4-triazol-3-ol

**DOI:** 10.1107/S1600536809001482

**Published:** 2009-01-17

**Authors:** Muhammad Hanif, Ghulam Qadeer, Nasim Hasan Rama, Javeed Akhtar, Madeleine Helliwell

**Affiliations:** a Department of Chemistry, Quaid-I-Azam Univeristy, Islamabad 45320, Pakistan; b The Manchester Materials Science Centre and Department of Chemistry, University of Manchester, Oxford Road, Manchester M13 9PL, England

## Abstract

In the mol­ecule of the title compound, C_17_H_17_N_3_O_3_, the triazole ring is oriented at dihedral angles of 88.09 (3) and 83.72 (3)° with respect to the 2-methoxy­benzyl and 2-methoxy­phenyl rings, respectively. The dihedral angle between the 2-methoxy­benzyl and 2-methoxy­phenyl rings is 52.95 (3)°. In the crystal structure, inter­molecular N—H⋯O hydrogen bonds link the mol­ecules into centrosymmetric dimers. There is a π–π contact between the 2-methoxy­phenyl rings [centroid–centroid distance = 3.811 (3) Å].

## Related literature

For general background, see: Demirbas *et al.* (2002 ▶); Holla *et al.* (1998 ▶); Kritsanida *et al.* (2002 ▶); Omar *et al.* (1986 ▶); Paulvannan *et al.* (2000 ▶); Turan-Zitouni *et al.* (1999 ▶). For related structures, see: Öztürk *et al.* (2004*a* ▶,*b* ▶). For bond-length data, see: Allen *et al.* (1987 ▶).
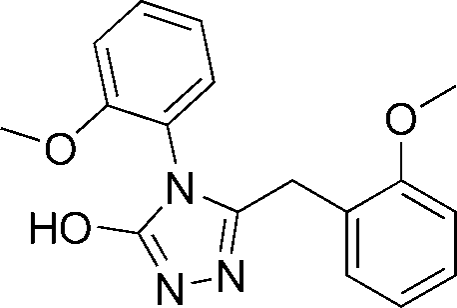


## Experimental

### 

#### Crystal data

C_17_H_17_N_3_O_3_
*M* *_r_* = 311.34
Monoclinic, 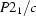

*a* = 7.4941 (12) Å
*b* = 8.3730 (13) Å
*c* = 24.770 (4) Å
β = 97.455 (2)°
*V* = 1541.2 (4) Å^3^
*Z* = 4
Mo *K*α radiation
μ = 0.09 mm^−1^
*T* = 100 (2) K
0.50 × 0.40 × 0.30 mm

#### Data collection

Bruker SMART CCD area-detector diffractometer
Absorption correction: none
11912 measured reflections
3160 independent reflections
2888 reflections with *I* > 2σ(*I*)
*R*_int_ = 0.039

#### Refinement

*R*[*F* ^2^ > 2σ(*F* ^2^)] = 0.037
*wR*(*F* ^2^) = 0.091
*S* = 1.06
3160 reflections
214 parameters
H atoms treated by a mixture of independent and constrained refinement
Δρ_max_ = 0.28 e Å^−3^
Δρ_min_ = −0.17 e Å^−3^

### 

Data collection: *SMART* (Bruker, 2001 ▶); cell refinement: *SAINT* (Bruker, 2002 ▶); data reduction: *SAINT*; program(s) used to solve structure: *SHELXS97* (Sheldrick, 2008 ▶); program(s) used to refine structure: *SHELXL97* (Sheldrick, 2008 ▶); molecular graphics: *SHELXTL* (Sheldrick, 2008 ▶); software used to prepare material for publication: *SHELXTL*.

## Supplementary Material

Crystal structure: contains datablocks I, global. DOI: 10.1107/S1600536809001482/hk2613sup1.cif

Structure factors: contains datablocks I. DOI: 10.1107/S1600536809001482/hk2613Isup2.hkl

Additional supplementary materials:  crystallographic information; 3D view; checkCIF report

## Figures and Tables

**Table 1:** Hydrogen-bond geometry (Å, °)

*D*—H⋯*A*	*D*—H	H⋯*A*	*D*⋯*A*	*D*—H⋯*A*
N3—H3*N*⋯O1^i^	0.890 (17)	1.911 (18)	2.7958 (14)	172.6 (15)

Symmetry code: (i) 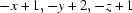.

**Fig. 1.**
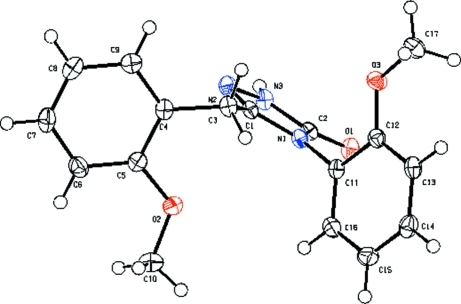
The molecular structure of the title molecule, with the atom-numbering scheme. Displacement ellipsoids are drawn at the 50% probability level.

**Fig. 2.**
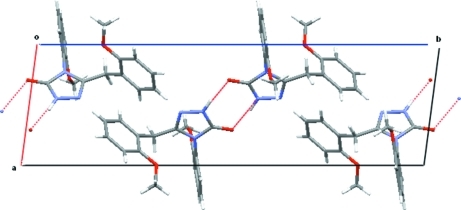
A partial packing diagram. Hydrogen bonds are shown as dashed lines.

**Fig. 3.**
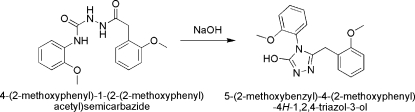
Reaction scheme.

## supplementary crystallographic information

### Comment

Substituted triazole derivatives display significant biological activities including antimicrobial (Holla *et al.*,  1998), analgesic (Turan-Zitouni *et al.*, 1999), antitumor (Demirbas *et al.*,  2002), antihypertensive (Paulvannan *et al.*,  2000) and antiviral (Kritsanida *et al.*,  2002) activities. The biological activity is closely related to the structure, possibly being due to the presence of the —N—C—S unit (Omar *et al.*,  1986). We are interested in the syntheses and biological activities of the aryloxyacetyl hydrazide derivatives and report herein the synthesis and crystal structure of the title compound.

In the molecule of the title compound (Fig. 1), the bond lengths (Allen *et al.*,  1987) and angles are within normal ranges, and they are comparable with those observed in related structures (Öztürk *et al.*, 2004*a*,b). In the triazole ring, the N2=C1 [1.2960 (16) Å] bond has double bond character. Rings A (N1/N2/N3/C1/C2), B (C4-C9) and C (C11-C16) are, of course, planar and the dihedral angles between them are A/B = 88.09 (3)°, A/C = 83.72 (3)° and B/C = 52.95 (3)°.

In the crystal structure, intermolecular N-H···O hydrogen bonds (Table 1) link the molecules into centrosymmetric dimers (Fig. 2), in which they may be effective in the stabilization of the structure. The π—π contact between the 2-methoxyphenyl rings, Cg1···Cg1^i^ [symmetry code: (i) -x, 2 - y, -z, where Cg1 is the centroid of the ring C (C11-C16)] may further stabilize the structure, with centroid-centroid distance of 3.811 (3) Å.

### Experimental

The synthesis of the title compound was carried out by refluxing a solution of 4-(2-methoxyphenyl)-1-(2-(2-methoxyphenyl)acetyl)semicarbazide (3.29 g, 10 mmol) in NaOH (2M) for 5 h. Crystals suitable for X-ray analysis were obtained by recrystallization from an aqeous ethanol solution at room temperature (yield; 79%; m.p. 444–445 K).

### Refinement

H3N atom (for NH) was located in difference synthesis and refined isotropically [N-H = 0.890 (17) Å and U_iso_(H) = 0.029 (4) Å^2^]. The remaining H atoms were positioned geometrically, with C-H = 0.95, 0.99 and 0.98 Å for aromatic, methylene and methyl H, respectively, and constrained to ride on their parent atoms with U_iso_(H) = xU_eq_(C), where x = 1.5 for methyl H and x = 1.2 for all other H atoms.

### Crystal data

**Table:**

C_17_H_17_N_3_O_3_	*F*(000) = 656
*M**_r_* = 311.34	*D*_x_ = 1.342 Mg m^−^^3^
Monoclinic, *P*2_1_/*c*	Melting point: 444(1) K
Hall symbol: -P 2ybc	Mo *K*α radiation, λ = 0.71073 Å
*a* = 7.4941 (12) Å	Cell parameters from 912 reflections
*b* = 8.3730 (13) Å	θ = 2.7–26.4°
*c* = 24.770 (4) Å	µ = 0.09 mm^−^^1^
β = 97.455 (2)°	*T* = 100 K
*V* = 1541.2 (4) Å^3^	Block, colorless
*Z* = 4	0.50 × 0.40 × 0.30 mm

### Data collection

**Table:**

Bruker SMART CCD area-detector diffractometer	2888 reflections with *I* > 2σ(*I*)
Radiation source: fine-focus sealed tube	*R*_int_ = 0.039
graphite	θ_max_ = 26.4°, θ_min_ = 2.6°
φ and ω scans	*h* = −9→9
11912 measured reflections	*k* = −10→10
3160 independent reflections	*l* = −30→30

### Refinement

**Table:**

Refinement on *F*^2^	Primary atom site location: structure-invariant direct methods
Least-squares matrix: full	Secondary atom site location: difference Fourier map
*R*[*F*^2^ > 2σ(*F*^2^)] = 0.037	Hydrogen site location: inferred from neighbouring sites
*wR*(*F*^2^) = 0.091	H atoms treated by a mixture of independent and constrained refinement
*S* = 1.06	*w* = 1/[σ^2^(*F*_o_^2^) + (0.0403*P*)^2^ + 0.586*P*] where *P* = (*F*_o_^2^ + 2*F*_c_^2^)/3
3160 reflections	(Δ/σ)_max_ = 0.001
214 parameters	Δρ_max_ = 0.28 e Å^−^^3^
0 restraints	Δρ_min_ = −0.17 e Å^−^^3^

### Special details

**Table:**

Geometry. All e.s.d.'s (except the e.s.d. in the dihedral angle between two l.s. planes) are estimated using the full covariance matrix. The cell e.s.d.'s are taken into account individually in the estimation of e.s.d.'s in distances, angles and torsion angles; correlations between e.s.d.'s in cell parameters are only used when they are defined by crystal symmetry. An approximate (isotropic) treatment of cell e.s.d.'s is used for estimating e.s.d.'s involving l.s. planes.
Refinement. Refinement of *F*^2^ against ALL reflections. The weighted *R*-factor *wR* and goodness of fit *S* are based on *F*^2^, conventional *R*-factors *R* are based on *F*, with *F* set to zero for negative *F*^2^. The threshold expression of *F*^2^ > σ(*F*^2^) is used only for calculating *R*-factors(gt) *etc*. and is not relevant to the choice of reflections for refinement. *R*-factors based on *F*^2^ are statistically about twice as large as those based on *F*, and *R*- factors based on ALL data will be even larger.

### Fractional atomic coordinates and isotropic or equivalent isotropic displacement parameters (Å^2^)

**Table:**

	*x*	*y*	*z*	*U*_iso_*/*U*_eq_	
O1	0.29433 (12)	0.86489 (10)	0.48842 (3)	0.0211 (2)	
O2	0.00410 (12)	1.09258 (11)	0.66422 (4)	0.0245 (2)	
O3	0.28378 (12)	0.51980 (10)	0.59145 (4)	0.0232 (2)	
N1	0.22479 (14)	0.83390 (12)	0.57791 (4)	0.0170 (2)	
N2	0.43854 (14)	0.98640 (12)	0.62165 (4)	0.0191 (2)	
N3	0.44636 (14)	0.98655 (12)	0.56595 (4)	0.0186 (2)	
H3N	0.533 (2)	1.0380 (19)	0.5514 (7)	0.029 (4)*	
C1	0.30443 (16)	0.89290 (14)	0.62733 (5)	0.0171 (2)	
C2	0.32018 (16)	0.89290 (14)	0.53796 (5)	0.0171 (3)	
C3	0.24258 (17)	0.84679 (15)	0.68016 (5)	0.0198 (3)	
H3A	0.3194	0.7587	0.6966	0.024*	
H3B	0.1177	0.8061	0.6729	0.024*	
C4	0.24810 (16)	0.98237 (14)	0.72050 (5)	0.0186 (3)	
C5	0.12083 (16)	1.10515 (15)	0.71152 (5)	0.0200 (3)	
C6	0.11769 (18)	1.22880 (16)	0.74885 (5)	0.0244 (3)	
H6	0.0328	1.3131	0.7421	0.029*	
C7	0.23959 (19)	1.22812 (17)	0.79612 (5)	0.0265 (3)	
H7	0.2370	1.3118	0.8219	0.032*	
C8	0.36460 (18)	1.10676 (17)	0.80600 (5)	0.0253 (3)	
H8	0.4467	1.1061	0.8386	0.030*	
C9	0.36928 (17)	0.98536 (16)	0.76775 (5)	0.0221 (3)	
H9	0.4570	0.9033	0.7742	0.027*	
C10	−0.15902 (18)	1.18333 (18)	0.66085 (6)	0.0296 (3)	
H10A	−0.2236	1.1557	0.6915	0.044*	
H10B	−0.2348	1.1588	0.6266	0.044*	
H10C	−0.1303	1.2976	0.6622	0.044*	
C11	0.07604 (16)	0.72543 (14)	0.56880 (5)	0.0170 (3)	
C12	0.10903 (16)	0.56170 (14)	0.57578 (5)	0.0185 (3)	
C13	−0.03451 (18)	0.45580 (15)	0.56618 (5)	0.0218 (3)	
H13	−0.0146	0.3441	0.5702	0.026*	
C14	−0.20681 (18)	0.51395 (16)	0.55077 (5)	0.0245 (3)	
H14	−0.3045	0.4412	0.5442	0.029*	
C15	−0.23904 (17)	0.67620 (17)	0.54470 (6)	0.0253 (3)	
H15	−0.3580	0.7145	0.5345	0.030*	
C16	−0.09601 (17)	0.78260 (16)	0.55360 (5)	0.0217 (3)	
H16	−0.1164	0.8941	0.5492	0.026*	
C17	0.3195 (2)	0.35431 (15)	0.60309 (6)	0.0278 (3)	
H17A	0.2530	0.3200	0.6326	0.042*	
H17B	0.4488	0.3392	0.6142	0.042*	
H17C	0.2813	0.2906	0.5704	0.042*	

### Atomic displacement parameters (Å^2^)

**Table:**

	*U*^11^	*U*^22^	*U*^33^	*U*^12^	*U*^13^	*U*^23^
O1	0.0264 (5)	0.0198 (4)	0.0175 (4)	−0.0037 (4)	0.0046 (4)	−0.0005 (3)
O2	0.0214 (5)	0.0280 (5)	0.0231 (5)	0.0027 (4)	−0.0005 (4)	−0.0034 (4)
O3	0.0224 (5)	0.0155 (4)	0.0309 (5)	0.0022 (3)	0.0004 (4)	0.0022 (4)
N1	0.0198 (5)	0.0142 (5)	0.0177 (5)	0.0001 (4)	0.0048 (4)	0.0000 (4)
N2	0.0221 (5)	0.0177 (5)	0.0179 (5)	−0.0004 (4)	0.0045 (4)	−0.0004 (4)
N3	0.0218 (5)	0.0170 (5)	0.0180 (5)	−0.0029 (4)	0.0066 (4)	0.0000 (4)
C1	0.0195 (6)	0.0131 (6)	0.0189 (6)	0.0021 (4)	0.0034 (5)	−0.0008 (4)
C2	0.0189 (6)	0.0127 (6)	0.0202 (6)	0.0021 (4)	0.0047 (5)	0.0008 (4)
C3	0.0236 (6)	0.0175 (6)	0.0190 (6)	−0.0016 (5)	0.0062 (5)	−0.0001 (5)
C4	0.0206 (6)	0.0187 (6)	0.0176 (6)	−0.0042 (5)	0.0070 (5)	0.0000 (5)
C5	0.0187 (6)	0.0232 (6)	0.0186 (6)	−0.0027 (5)	0.0048 (5)	−0.0004 (5)
C6	0.0234 (6)	0.0236 (7)	0.0274 (7)	0.0016 (5)	0.0076 (5)	−0.0034 (5)
C7	0.0304 (7)	0.0265 (7)	0.0238 (7)	−0.0056 (6)	0.0079 (6)	−0.0088 (5)
C8	0.0260 (7)	0.0298 (7)	0.0198 (6)	−0.0076 (5)	0.0018 (5)	−0.0014 (5)
C9	0.0209 (6)	0.0237 (7)	0.0221 (6)	−0.0013 (5)	0.0045 (5)	0.0024 (5)
C10	0.0217 (7)	0.0337 (8)	0.0326 (8)	0.0036 (6)	0.0007 (6)	0.0025 (6)
C11	0.0201 (6)	0.0164 (6)	0.0153 (6)	−0.0029 (5)	0.0050 (5)	−0.0011 (4)
C12	0.0215 (6)	0.0183 (6)	0.0160 (6)	0.0009 (5)	0.0036 (5)	−0.0002 (5)
C13	0.0280 (7)	0.0168 (6)	0.0214 (6)	−0.0029 (5)	0.0059 (5)	−0.0014 (5)
C14	0.0234 (6)	0.0262 (7)	0.0246 (7)	−0.0080 (5)	0.0062 (5)	−0.0047 (5)
C15	0.0190 (6)	0.0302 (7)	0.0267 (7)	0.0021 (5)	0.0028 (5)	−0.0031 (5)
C16	0.0252 (7)	0.0192 (6)	0.0211 (6)	0.0030 (5)	0.0043 (5)	−0.0009 (5)
C17	0.0322 (7)	0.0172 (7)	0.0328 (7)	0.0055 (5)	0.0003 (6)	0.0029 (5)

### Geometric parameters (Å, °)

**Table:**

O1—C2	1.2397 (15)	C7—C8	1.382 (2)
O2—C5	1.3724 (15)	C7—H7	0.9500
O2—C10	1.4326 (16)	C8—C9	1.3931 (19)
O3—C12	1.3627 (15)	C8—H8	0.9500
O3—C17	1.4335 (15)	C9—H9	0.9500
N1—C1	1.3817 (16)	C10—H10A	0.9800
N1—C2	1.3848 (15)	C10—H10B	0.9800
N1—C11	1.4329 (15)	C10—H10C	0.9800
N2—C1	1.2960 (16)	C11—C16	1.3811 (18)
N2—N3	1.3884 (15)	C11—C12	1.3998 (17)
N3—C2	1.3489 (16)	C12—C13	1.3905 (18)
N3—H3N	0.890 (17)	C13—C14	1.3865 (19)
C1—C3	1.4947 (17)	C13—H13	0.9500
C3—C4	1.5093 (17)	C14—C15	1.385 (2)
C3—H3A	0.9900	C14—H14	0.9500
C3—H3B	0.9900	C15—C16	1.3892 (19)
C4—C9	1.3854 (18)	C15—H15	0.9500
C4—C5	1.4004 (18)	C16—H16	0.9500
C5—C6	1.3903 (18)	C17—H17A	0.9800
C6—C7	1.3887 (19)	C17—H17B	0.9800
C6—H6	0.9500	C17—H17C	0.9800
			
C5—O2—C10	116.97 (10)	C9—C8—H8	120.3
C12—O3—C17	116.99 (10)	C4—C9—C8	121.08 (12)
C1—N1—C2	107.57 (10)	C4—C9—H9	119.5
C1—N1—C11	127.15 (10)	C8—C9—H9	119.5
C2—N1—C11	125.22 (10)	O2—C10—H10A	109.5
C1—N2—N3	103.99 (10)	O2—C10—H10B	109.5
C2—N3—N2	113.14 (10)	H10A—C10—H10B	109.5
C2—N3—H3N	124.8 (10)	O2—C10—H10C	109.5
N2—N3—H3N	121.9 (10)	H10A—C10—H10C	109.5
N2—C1—N1	111.82 (11)	H10B—C10—H10C	109.5
N2—C1—C3	125.74 (11)	C16—C11—C12	121.12 (11)
N1—C1—C3	122.41 (11)	C16—C11—N1	120.19 (11)
O1—C2—N3	128.89 (11)	C12—C11—N1	118.68 (11)
O1—C2—N1	127.66 (11)	O3—C12—C13	125.32 (11)
N3—C2—N1	103.45 (10)	O3—C12—C11	115.79 (11)
C1—C3—C4	113.60 (10)	C13—C12—C11	118.89 (11)
C1—C3—H3A	108.8	C14—C13—C12	119.69 (12)
C4—C3—H3A	108.8	C14—C13—H13	120.2
C1—C3—H3B	108.8	C12—C13—H13	120.2
C4—C3—H3B	108.8	C15—C14—C13	121.17 (12)
H3A—C3—H3B	107.7	C15—C14—H14	119.4
C9—C4—C5	118.63 (12)	C13—C14—H14	119.4
C9—C4—C3	122.11 (11)	C14—C15—C16	119.46 (12)
C5—C4—C3	119.17 (11)	C14—C15—H15	120.3
O2—C5—C6	124.12 (12)	C16—C15—H15	120.3
O2—C5—C4	115.15 (11)	C11—C16—C15	119.65 (12)
C6—C5—C4	120.73 (12)	C11—C16—H16	120.2
C7—C6—C5	119.45 (12)	C15—C16—H16	120.2
C7—C6—H6	120.3	O3—C17—H17A	109.5
C5—C6—H6	120.3	O3—C17—H17B	109.5
C8—C7—C6	120.59 (12)	H17A—C17—H17B	109.5
C8—C7—H7	119.7	O3—C17—H17C	109.5
C6—C7—H7	119.7	H17A—C17—H17C	109.5
C7—C8—C9	119.49 (12)	H17B—C17—H17C	109.5
C7—C8—H8	120.3		
			
C1—N2—N3—C2	−0.75 (13)	C4—C5—C6—C7	−1.53 (19)
N3—N2—C1—N1	−0.47 (13)	C5—C6—C7—C8	0.7 (2)
N3—N2—C1—C3	177.59 (11)	C6—C7—C8—C9	0.8 (2)
C2—N1—C1—N2	1.47 (13)	C5—C4—C9—C8	0.56 (19)
C11—N1—C1—N2	178.77 (11)	C3—C4—C9—C8	−176.01 (12)
C2—N1—C1—C3	−176.66 (11)	C7—C8—C9—C4	−1.4 (2)
C11—N1—C1—C3	0.64 (18)	C1—N1—C11—C16	97.89 (15)
N2—N3—C2—O1	−178.27 (12)	C2—N1—C11—C16	−85.26 (15)
N2—N3—C2—N1	1.60 (13)	C1—N1—C11—C12	−81.92 (15)
C1—N1—C2—O1	178.09 (12)	C2—N1—C11—C12	94.93 (14)
C11—N1—C2—O1	0.72 (19)	C17—O3—C12—C13	−5.25 (18)
C1—N1—C2—N3	−1.78 (12)	C17—O3—C12—C11	175.20 (11)
C11—N1—C2—N3	−179.14 (11)	C16—C11—C12—O3	−179.25 (11)
N2—C1—C3—C4	39.80 (17)	N1—C11—C12—O3	0.56 (16)
N1—C1—C3—C4	−142.34 (11)	C16—C11—C12—C13	1.16 (18)
C1—C3—C4—C9	−111.02 (13)	N1—C11—C12—C13	−179.03 (11)
C1—C3—C4—C5	72.42 (15)	O3—C12—C13—C14	179.51 (11)
C10—O2—C5—C6	−18.72 (18)	C11—C12—C13—C14	−0.95 (18)
C10—O2—C5—C4	161.34 (11)	C12—C13—C14—C15	0.01 (19)
C9—C4—C5—O2	−179.15 (11)	C13—C14—C15—C16	0.8 (2)
C3—C4—C5—O2	−2.47 (16)	C12—C11—C16—C15	−0.41 (19)
C9—C4—C5—C6	0.92 (18)	N1—C11—C16—C15	179.78 (11)
C3—C4—C5—C6	177.59 (11)	C14—C15—C16—C11	−0.54 (19)
O2—C5—C6—C7	178.54 (12)		

### Hydrogen-bond geometry (Å, °)

**Table:**

*D*—H···*A*	*D*—H	H···*A*	*D*···*A*	*D*—H···*A*
N3—H3N···O1^i^	0.890 (17)	1.911 (18)	2.7958 (14)	172.6 (15)

Symmetry codes: (i) −*x*+1, −*y*+2, −*z*+1.
